# Y-chromosome evidence supports widespread signatures of three-species *Canis* hybridization in eastern North America

**DOI:** 10.1002/ece3.301

**Published:** 2012-08-13

**Authors:** Paul J Wilson, Linda Y Rutledge, Tyler J Wheeldon, Brent R Patterson, Bradley N White

**Affiliations:** 1 Natural Resources DNA Profiling & Forensic Centre, Trent UniversityDNA Building, 2140 East Bank Drive, Peterborough, Ontario, Canada, K9J 7B8; 2 Environmental & Life Sciences Graduate Program, Trent UniversityDNA Building, 2140 East Bank Drive, Peterborough, Ontario, Canada, K9J 7B8; 3 Ontario Ministry of Natural Resources, Trent UniversityDNA Building, 2140 East Bank Drive, Peterborough, Ontario, Canada, K9J 7B8

**Keywords:** *Canis*, eastern wolf, hybridization, microsatellites, Y-chromosome, Y-intron

## Abstract

There has been considerable discussion on the origin of the red wolf and eastern wolf and their evolution independent of the gray wolf. We analyzed mitochondrial DNA (mtDNA) and a Y-chromosome intron sequence in combination with Y-chromosome microsatellites from wolves and coyotes within the range of extensive wolf–coyote hybridization, that is, eastern North America. The detection of divergent Y-chromosome haplotypes in the historic range of the eastern wolf is concordant with earlier mtDNA findings, and the absence of these haplotypes in western coyotes supports the existence of the North American evolved eastern wolf (*Canis lycaon*). Having haplotypes observed exclusively in eastern North America as a result of insufficient sampling in the historic range of the coyote or that these lineages subsequently went extinct in western geographies is unlikely given that eastern-specific mtDNA and Y-chromosome haplotypes represent lineages divergent from those observed in extant western coyotes. By combining Y-chromosome and mtDNA distributional patterns, we identified hybrid genomes of eastern wolf, coyote, gray wolf, and potentially dog origin in *Canis* populations of central and eastern North America. The natural contemporary eastern *Canis* populations represent an important example of widespread introgression resulting in hybrid genomes across the original *C. lycaon* range that appears to be facilitated by the eastern wolf acting as a conduit for hybridization. Applying conventional taxonomic nomenclature and species-based conservation initiatives, particularly in human-modified landscapes, may be counterproductive to the effective management of these hybrids and fails to consider their evolutionary potential.

## Introduction

Elucidating the taxonomic relationships and evolutionary origin of North American “*Canis*” has been controversial, with considerable discussion over the number of contemporary wolf species. Originally centered on the red wolf (*Canis rufus*) (Nowak 1979; Wayne and Jenks 1991; Roy et al. 1994; Nowak et al. 1998; Wayne et al. 1998), the controversy has been extended to the eastern wolf (*Canis lycaon*) (e.g., Koblmüller et al. 2009; Fain et al. 2010). Both species have been identified as smaller wolves that readily hybridize with coyotes. Initial genetic studies proposed an origin of red and eastern wolves through gray wolf (*C. lupus*) and western coyote (*Canis latrans*) hybridization based on a lack of distinct genetic material (Wayne and Jenks 1991; Roy et al. 1994). More recent genetic analyses, however, identified distinct mitochondrial DNA (mtDNA) that supports a North American evolution of the eastern wolf (Rutledge et al. 2010a, 2010b). The debate over the number of North American wolf species has been confounded by various proposed hybridization scenarios (Leonard and Wayne 2008; Koblmüller et al. 2009; Wheeldon and White 2009; Wilson et al. 2009; Wheeldon et al. 2010; vonHoldt et al. 2011). Interestingly, extensive *Canis* hybridization appears limited to the historic distribution of eastern wolves and red wolves (i.e., primarily east of the Mississippi River within the eastern temperate forests, which probably included Wisconsin and Michigan) with notable limitations to hybridization in more western geographies, particularly between coyotes and gray wolves (Pilgrim et al. 1998; Leonard et al. 2005; Hailer and Leonard 2008).

The difficulty with interpreting the evolutionary history of *Canis* using mtDNA is that hybridization between eastern wolves (see Figure S1) and coyotes would have caused introgression of closely related sequences from a proposed common New World lineage, both recently (Wilson et al. 2000, 2009) and potentially historically (Wilson et al. 2003; Rutledge et al. 2010b). To test the hypothesis that the eastern wolf, that includes the red wolf for the purpose of this study, evolved in eastern North America independent of the gray wolf, and that it is more closely related to the coyote (Wilson et al. 2000, 2003), we assessed the geographic distribution of paternally inherited Y-chromosomes in male wolves and coyotes in combination with previously described mtDNA sequences proposed to originate from the eastern wolf (Wilson et al. 2000; Wheeldon and White 2009; Rutledge et al. 2010a). This approach has been applied to a regional study in Texas that described localized hybridization among three historically sympatric species: the gray wolf, the coyote, and the red wolf (Hailer and Leonard 2008). That study identified species-specific Y-chromosome microsatellite alleles for gray wolves and coyotes, but it did not consider the relationship of the eastern wolf in the larger *Canis* evolutionary model and it did not consider the Y-intron sequences in association with the Y-microsatellite haplotypes. Here, we provide novel analysis of Y-intron sequences in conjunction with Y-chromosome microsatellite alleles across a wide geographic range to test the hypothesis of a distinct eastern wolf paternal lineage. We predicted that a North American evolved wolf would have evolved Y-chromosome haplotypes, concordant with previously published mtDNA results (Wilson et al. 2000, 2003; Rutledge et al. 2010a), that were divergent from gray wolves and coyotes and that were geographically localized to the historic distribution of *C. lycaon* (i.e., in general, east of the Mississippi River within the eastern temperate forest region). As a result of extensive levels of hybridization, these species-specific DNA markers would persist in current eastern *Canis* hybrids, but would be absent from nonhybridizing western coyotes.

## Materials and Methods

We extracted DNA from *Canis* samples (see Table 2 for sample sizes) using a DNeasy Blood & Tissue Kit (Qiagen Inc., Mississauga, Ontario, Canada). Samples were collected under capture and handling procedures approved by the Ontario Ministry of Natural Resources' animal care committee or were submitted by registered hunters and trappers. Red wolf samples were provided by the red wolf captive breeding program.

Sex was determined by amplification of the last intron of the *Zfx/Zfy* genes (Shaw et al. 2003). Confirmed males were then profiled at four Y-chromosome microsatellite loci (MS34A, MS34B, MS41A, and MS41B) (Sundqvist et al. 2001) and at a 658 bp fragment of the *Zfy* intron with primers LGL-331 (5′-CAA ATC ATG CAA GGA TAG AC-3′) and Yint2-335 (5′-GTC CAT TGG ATA ATT CTT TCC-3′; Shaw et al. 2003). The polymerase chain reaction (PCR), chemical and cycling conditions for the Y-chromosome microsatellite loci were as follows: For MS34, 5–10 ng of DNA was amplified in a 15 μL reaction with 1× PCR buffer, 0.2 mm dNTPs (Invitrogen, Burlington, Ontario, Canada), 1.5 mm MgCl_2_, 0.1 μm MS34A-F primer, 0.15 μm MS34B-F primer, 0.2 μm MS34-R primer, and 1 U *Taq* DNA polymerase (Invitrogen). PCR cycling included an initial denaturation at 94°C for 5 min followed by 30 cycles of 94°C for 30 sec, annealing at 60°C for 1 min, and extension at 72°C for 1 min, with a final cycle of 60°C for 45 min and storage at 4°C. Conditions for MS41 were similar to MS34, except that primer concentrations were 0.15 μm MS41A-F primer, 0.2 μm MS41B-F primer, 0.2 μm MS41-R primer, and the annealing temperature was 58°C. The Y-intron was amplified under the following PCR conditions in a 20 μL reaction: approximately 5–10 ng of DNA, 1× PCR buffer, 0.2 mm dNTPs, 1.5 mm MgCl_2_, 0.2 mm each primer, 0.1 μg bovine serum albumin, and 1 U *Taq* DNA polymerase. PCR steps included initial denaturation at 94°C for 5 min followed by 35 cycles of 94°C for 30 sec, 52°C for 30 sec, and 72°C for 30 sec, followed by a final extension at 72°C for 10 min. All sequencing and microsatellite fragment separation and visualization were performed on a MegaBACE 1000 (GE Healthcare, Baie d'Urfé, Quebec, Canada).

Composite haplotypes were determined based on the alleles present at the four loci. Y-microsatellite haplotypes were standardized to previously published data (Hailer and Leonard 2008) (Table S1). We generated a 400 bp sequence of the last *Zfy* intron for each microsatellite Y-haplotype. Sequences of the mtDNA control region were generated with primers and conditions previously described in Wilson et al. (2000, 2003). In total, we analyzed 364 wolves and coyotes (Table 1) plus an additional 71 coyotes from previously published literature (Table 2) at the Y-chromosome (Table 1), and 718 wolves and coyotes at the mtDNA control region plus an additional 124 coyotes from previously published literature (Table 2). We used the Y-intron data in combination with the Y-microsatellite data to generate a median-joining network in NETWORK v.4.516 (http://www.fluxus-engineering.com) using methods described in Bandelt et al. (1999) and using nucleotide states to describe the *Zfy* intron sequence variation with the microsatellite allele haplotype combinations. A 2:1 weighting was assigned to transversions over transition site differences for the *Zfy* intron, and the intron sequence variation was weighted twice as high as microsatellite loci. Nucleotide diversity (Pi) of the four *Zfy* intron sequences was estimated using the software DnaSP v5 (Librado and Rozas 2009). We used the prop.test function in R 2.13.1 (R Development Core Team 2011) to test the null hypothesis that the frequency of the putative eastern wolf Y-chromosome haplotypes associated with *Zfy-*4 was the same in western coyotes (0/121) as observed in eastern *Canis* populations (90/288) (see Table 2).

**Table 1:** Summary of sampled regions including the number of individuals (N) and frequency of occurrence of Y-chromosome haplotypes (in brackets) per geographic region

Region	N	Haplotypes
Northwest territories (W)	26	2AF(7), 2CC(3), 2CE(6), 2CF(1), 2CG(3), 2CT(2), 2DC(2), 2EF(2)
Manitoba (W)	20	2AF(7), 2AT(1), 2CE(5), 2DC(6), 4BB(1)
Northwestern Ontario (W)	18	2AF(2), 2CC(2), 2CE(2), 2CS(3), 4AA(1), 4BB(8)
Northeastern Ontario (W)	26	2AF(4), 2CE(8), 2CF(5), 2CT(8), 4BB(1)
Quebec (W)	13	2CC(2), 2CE(2), 2CF(3), 2CS(3), 4AA(1), 4BB(2)
Algonquin Park (W+C)	53	1CD(2), 1CR(1), 1GP(1), 2CE(2), 2CG(1), 2CS(3), 2EF(3), 4AA(26), 4BB(14)
Southeastern Ontario (C)	37	1CD(8), 1CI (1), 1GP (3), 2CE(2), 2FL(1), 2HS (1), 2HT(1), 4AA(18), 4BB (2)
New York (C)	33	1CD(18), 1GP(3), 2CF(1), 2FF(8), 2HT(2), 4AA(1)
Maine/New Brunswick (C)	38	1CD(7), 1GP(6), 2FF(4), 2FG(5), 2FL(8), 4AA(8)
North Carolina (C)	11	1CI(4), 1CM(4), 2HG(1), 2HS(1), 4BR(1)
Texas (C)	15	1CP(2), 3EA(2), 3EC(1), 3HI(1), 3HN(4), 3HO(2), 3HP(2), 3IQ(1)
Saskatchewan (C)	35	1AQ(1), 1CI(6), 1CK(4), 1CM(1), 1CN(7), 1CO(1), 1CQ(4), 1DQ(4), 3EA(2), 3EJ(1), 3EO(2), 3FA(2)
Louisiana (U)	14	1CM(2), 2FL(3), 2HS(2), 2HT(1), 4BB(4), 4BR(2)
Captive Red Wolves (W)	25	2FL(9), 3EA(16)

Haplotype codes correspond to the *Zfy* intron sequence followed by the allele letter designations for loci MS34 (first letter) and MS41 (second letter). Letter in brackets indicates if samples were from wolves (W), coyotes (C), or unknown (U). Unknown samples were collected from a fur house and had no species designation assigned. Reference to these samples as red wolves in the text is from the perspective of the original red wolf geographic range.

**Table 2:** Distribution of species-specific Y-chromosome microsatellite and mtDNA haplotypes in North American *Canis* specimens

	Y-chromosome						Mitochondrial DNA						
		
Region	Y-chr	Clu^1^	Cly^2^_AA_	Cly^2^_BB_	Cly^2^_BR_	Cla^3^	mtDNA	Clu^4^	Cly^5^_C1_	Cly^5^_C3_	Cly^6^_C13_	Cru^7^_C2_	Cla^8^
Northwest territories (W)	26	26					50	50					
Manitoba (W)	20	19	1				32	19		13			
NW Ontario (W)	18	9	1	8			33	10		16	6		1
NE Ontario (W)	26	25		1			51	27		1	6		17
Quebec (W)	13	10	1	2			26	9	6	1	1		9
Algonquin Park (W+C)	53	9	26	14		4	127	9	3	1	1		113
Southeastern Ontario (C)	37	5	18	2		12	99		18		2		79
New York (C)	33	11	1			21	53		19				34
Maine/New Brunswick (C)	38	17	8			13	81		32				49
North Carolina (C)	11	2			1	8	13	4**					9
Nebraska (C)*	37*					37	71*						71
Texas (C)*	15 + 34*	2				47	27 + 53*						80
Saskatchewan (C)	35					35	68						68
Louisiana (U)	14	6	4		2	2	25	2				8	15
Captive Red Wolves (W)	25	9				16	33					33	

Letter in brackets indicates if samples were from wolves (W) or coyotes (C) or unknown (U).

**Figure 1:**
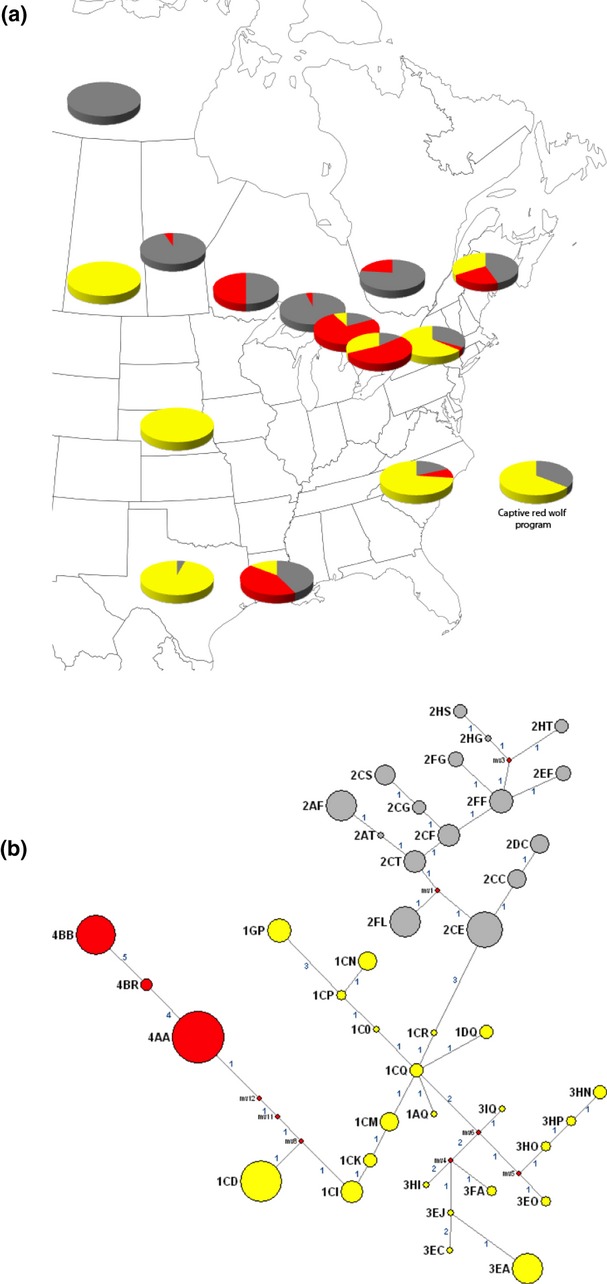
(a) Map of the distribution of North American *Canis* Y-chromosome haplotypes based on four microsatellite loci and an intron of the *Zfy* gene. Haplotypes are classified by species: gray for gray wolf (*C. lupus*); yellow for coyote (*C. latrans*); and red for eastern wolf (*C. lycaon*) origin. (b) Y-chromosome haplotype network classifying haplogroups as eastern wolf (red), coyote (yellow), and gray wolf (gray). The number is intron and letters are Y micros (refer to Supporting Information Table S1). The size of the node represents relative sample sizes and the number on the connections indicates the number of base pair repeat differences for the Y-specific microsatellite loci or nucleotide differences for the *Zfy* intron.

* Data from Hailer and Leonard (2008). The Y-haplotype with the gray wolf diagnostic allele (208 at locus MS41a) identified in a Texas coyote by Hailer and Leonard (2008) was also observed in this study, although this haplotype was linked with the coyote-specific intron-3 and not intron-2 diagnostic of gray wolves. This suggests a likely rare homoplasy. As a result, we have not graphed this haplotype as gray wolf-specific in Figure 1.

** Samples identified as *C. lupus* mtDNA, specifically dog, in Adams et al. (2003) and confirmed within our data set. Those regions in bold are considered coyotes (*n* = 121) from western regions (i.e., west of the Mississippi River).

1 Y-chromosome haplotypes containing the gray wolf (*C. lupus* [Clu]) diagnostic 208 allele at locus MS41a (Hailer and Leonard 2008) (although see above) and *Zfy* intron-2 identified in this study.

2 *C. lycaon* (Cly) Y-chromosome haplotypes containing the eastern-specific *Zfy* intron-4 identified in this study.

3 Y-chromosome haplotypes containing coyote (*C. latrans* [Cla]) specific alleles 212–218 at locus MS41a (Hailer and Leonard 2008) and *Zfy* intron-1 or -3 identified in this study.

4 Gray wolf (*C. lupus* [Clu]) mitochondrial DNA (mtDNA) haplotypes as identified in previous studies (Wilson et al. 2000, 2003).

5 Eastern wolf (*C. lycaon* [Cly]) mtDNA haplotypes identified in previous studies (Rutledge et al. 2010a, 2010b).

6 Putative eastern wolf (*C. lycaon* [Cly]) mtDNA haplotype based on the criteria of a coyote-like sequence (Wilson et al. 2003; Wheeldon and White 2009) with common frequency in wolves in eastern geographies, but absence in western coyotes. In contrast to some previous publications (Grewal et al. 2004; Rutledge et al. 2010c), here we consider C9 and C17 to be coyote sequences because there is sufficient disagreement at this time regarding their possible eastern wolf origin.

7 Putative red wolf (*C. rufus*) mtDNA haplotype identified in previous studies (Hailer and Leonard 2008), which we interpret as *C. lycaon* in origin based on criteria used for C13.

8 Coyote (*C. latrans* [Cla]) mtDNA haplotypes identified in previous studies (Wilson et al. 2000, 2003; Hailer and Leonard 2008).

## Results

Four different sequences were identified within 400 bp of the *Zfy* intron (*Zfy-1*, -*2*, -*3*, and -*4*; Genbank Accession numbers: FJ687618, FJ687619, JQ394817, and FJ687620) with three segregating sites. *Zfy-2*, *-3*, and *-4* each differed by one nucleotide from *Zfy-1*. *Zfy-2* was the only sequence found in Northwest Territories gray wolves (Table 1, Fig. 1a) and was associated with the specific Y-chromosome microsatellite allele size, previously identified as gray wolf (i.e., 208 at MS41a, [Hailer and Leonard 2008]). Intron-3 was observed in western coyotes and captive red wolves (Table 1) and was associated with the allele range identified as a coyote lineage (i.e., 212–218 at MS41a [Hailer and Leonard 2008], Table S1). *Zfy-1* haplotypes were found in western coyotes from Saskatchewan and Texas (Table 1, Table S1), and in eastern geographies (Table 1). *Zfy-4* was associated with Y-chromosome microsatellite alleles in the size range identified for coyotes, but this sequence was only found in the proposed historic range of the eastern wolf (including the red wolf, i.e., Louisiana), and was not observed in western coyotes (Table 1, Fig. 1a). These eastern wolf Y-chromosome haplotypes were found in 22% of eastern coyotes through southeastern Ontario and into the eastern United States (excluding Louisiana; Table 1, Fig. 1a). Also, gray wolf-like Y-chromosome haplotypes (associated with *Zfy-2*) were found in eastern coyotes throughout their range and in the captive red wolf population (Table 1, Fig. 1a). Overall nucleotide diversity per site (Pi) based on the four 400 bp sequences of the Y-intron was 0.00375 (±SD, 0.00091) and overall nucleotide divergence with Jukes–Cantor correction (K[JC]) was 0.00188. The average number of nucleotide substitutions per site (Dxy) for each intron sequence compared with *Zfy-1* was 0.0025. The proportion of *Zfy-*4 haplotypes in western coyotes was significantly lower than expected, based on the proportion of *Zfy-*4 introns found in eastern *Canis* populations (*P* = 8.371 × 10^–12^; 95% CI = 0.25–0.37).

**Figure 2:**
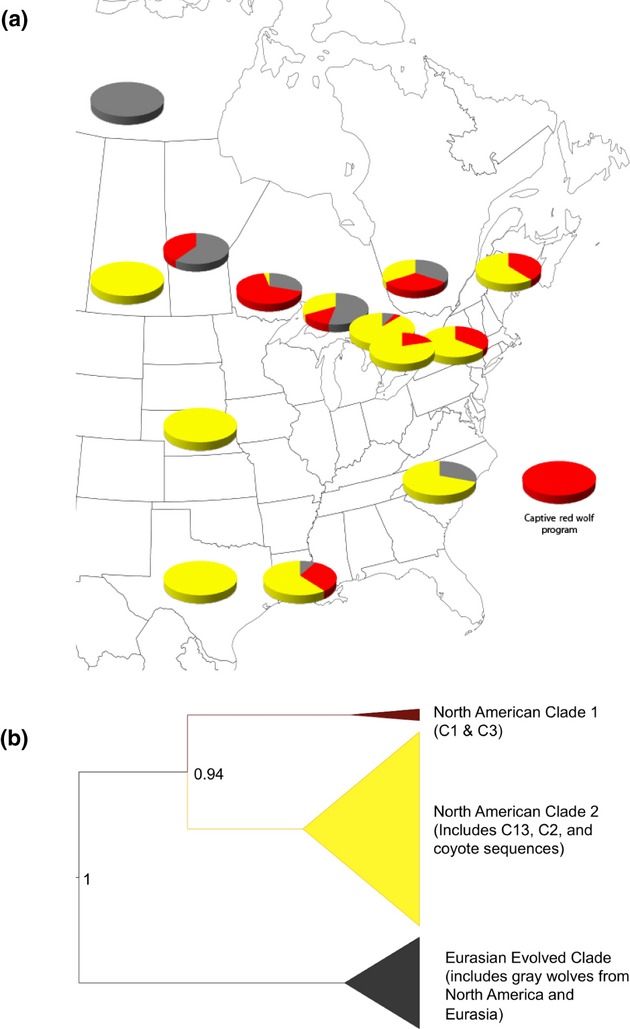
(a) Map of the distribution of North American *Canis* mitochondrial DNA control region haplotypes, classified by species: gray for gray wolf (*C. lupus*) (includes putative dog haplotypes); yellow for coyote (*C. latrans*); and red for eastern wolf (*C. lycaon*) origin. The haplotypes in red associated with the captive red wolf and Louisiana populations denote the red wolf sequence (C2), a putative *C. lycaon* haplotype. (b) A generalized mtDNA phylogenetic tree (adapted from Rutledge et al. [2010a] with permission) identifying the monophyletic C1 and C3, *C. lycaon* haplotypes and the putative *C. lycaon* haplotypes C2 and C13 grouping with coyote haplotypes. Branch values are Bayesian posterior probabilities.

The Y-chromosome network (Fig. 1b) shows clear distinctions between the haplogroups associated with coyotes (*Zfy-1* and *Zfy-3*), gray wolves (*Zfy-2*) and eastern wolves (*Zfy-4*) when incorporating *Zfy* intron sequences with Y-chromosome microsatellite haplotypes. The pattern of divergent eastern-specific Y-chromosomes is comparable with previously published phylogenetic analyses and geographic distribution of *Canis* mtDNA (Fig. 2a,b [Rutledge et al. 2010a]). Similar to the Y-chromosome patterns, there is a stark contrast in the mtDNA composition of western coyote populations compared with that of eastern *Canis* populations that contain *C. lycaon* mtDNA, specifically the reciprocally monophyletic clade that includes C1 and C3 (Fig. 2b). As noted elsewhere, mtDNA haplotypes C2 and C13 that group within the coyote clade are of possible *C. lycaon* origin because they are not found in western regions (Wheeldon and White 2009; Fain et al. 2010; Wheeldon et al. 2010). Three eastern wolf mtDNA haplotypes (C1, C3, and C13) occur in high frequency in the western Great Lakes states (Fain et al. 2010; Wheeldon et al. 2010) and/or Ontario, but they are absent in coyotes sampled from western populations (Table 2). C2 occurs in the captive red wolf population and has typically been identified as the red wolf haplotype (Hailer and Leonard 2008).

## Discussion

Here, we present new Y-chromosome intron sequence data and provide a novel network analysis of the intron haplotypes in connection with new and previously published Y-microsatellite haplotypes. We also connect the Y-chromosome data to a geographic distribution, and provide comparison of the Y-chromosome data with maternal *Canis* mtDNA haplotype lineages across a wide geographic distribution. The presence of eastern-specific Y-chromosome and mtDNA haplotypes, absent in nonhybridizing gray wolves and coyotes, supports the origin of a North American evolved eastern wolf. Both the branching patterns of the Y-chromosome and mtDNA suggest an independent divergent lineage of haplotypes closely associated with coyotes and distinct from gray wolves. However, both the monophyletic eastern wolf mtDNA clade (C1 and C3) and the eastern wolf Y-chromosome *Zfy*-4 haplotypes are clearly divergent from coyotes, and the *Zfy-*4 lineage appears to be as divergent from coyotes as coyotes are from gray wolves.

One potential alternative interpretation of these patterns is that these variant haplotypes represent coyote-specific maternal and paternal haplotypes within the overall variation in the species. The absence of these haplotypes in western geographies would then result from either localized extinctions due to genetic drift of those mtDNA and Y-chromosomes or a failure to sample them in the population. However, this explanation is highly improbable because the eastern-specific haplotypes are divergent at both the mtDNA and Y-chromosome. The likelihood of haplotype extinctions occurring in western coyotes independently twice for mtDNA (C1 and C3) and three times for the Y-chromosome (4AA, 4BB, 4BR) makes the alternative explanation of genetic drift statistically unlikely. Similarly, the geographic distribution of haplotypes could not be the result of recent coyote expansion within the last century because the timeframe is inconsistent with mutation rates of both markers.

Given the high frequency of mtDNA haplotypes C1, C3, and C13 in wolves from the western Great Lakes states (Fain et al. 2010; Wheeldon et al. 2010) and/or Ontario, and their absence from coyotes sampled from western populations (Table 2), it seems unlikely that these putative eastern wolf haplotypes would not have been detected in nonhybridizing coyotes. This criterion could also apply to the coyote-clade C2 haplotype found in the captive red wolf population (Hailer and Leonard 2008) and in the Louisiana population. Although loss of coyote-clustering sequences in western coyotes through random genetic drift following introgression cannot be ruled out, this scenario is much less likely for the eastern-specific monophyletic grouping of the C1 and C3 haplotypes (Wilson et al. 2000; Rutledge et al. 2010a). Although our study and previous studies have not provided a comprehensive survey of coyotes at mtDNA and Y-chromosomes farther to the west, evidence suggests the central US regions summarized in our study represents the core historical source of where coyotes expanded and colonized North America (Nowak 1979; Parker 1995). Additionally, the western coyote samples analyzed here (Texas, Nebraska, and Saskatchewan) are along the eastern front of recent coyote expansion and are even more likely to have similar haplotypes to those animals found within eastern regions.

Previously analyzed historical specimens further support the mtDNA haplotypes as having an eastern North America origin independent of gray wolves and western coyotes. MtDNA haplotypes observed in specimens collected from the mid-to-late 1800s in New York and Maine, prior to coyote colonization, had a C1 haplotype and a haplotype closely related to C13, thus excluding them as originating from gray wolves (*C. lupus*) (Wilson et al. 2003). This finding is consistent with the divergent eastern-specific haplotypes further characterized in this study. Although we cannot exclude the possibility of occasional pre-European introgressive hybridization between eastern wolves and coyotes, eastern-specific divergent mtDNA and Y-chromosome haplotypes originating from contemporary coyote expansion and colonization is highly unlikely.

Given the unlikelihood of alternative scenarios, we conclude that the data presented here further support the inclusion of two wolf species, in addition to coyotes, into interpretations of populations, such as the Great Lakes wolf (Wheeldon and White 2009; Fain et al. 2010; Wheeldon et al. 2010) and the eastern coyote (Kays et al. 2010). However, the extent of hybridization among *Canis* species is so prevalent in eastern North America that essentially all eastern populations of wolves and coyotes surveyed show evidence of mtDNA or Y-chromosome introgression. This includes the historic distribution of the red wolf, specifically the area in Texas where the red wolf animals used to breed the original founders were collected (Wayne and Jenks 1991) (as inferred from captive red wolves; see also Hailer and Leonard 2008). These animals may or may not contain eastern wolf mtDNA, depending on the origin of C2, and they lack eastern wolf Y-chromosomes. The evidence for limited direct *C. lupus* × *C. latrans* hybridization in western geographies (Pilgrim et al. 1998; Leonard et al. 2005) is further supported by the absence of gray wolf introgression into western coyotes that would have overlapped with declining gray wolf populations (Hailer and Leonard 2008). Ultimately, the lack of extensive hybridization in the west may reflect the eastern wolf's potential role as an intermediate conduit for mixing of coyote and gray wolf genomes with its own at a significantly broader regional and taxonomic scale than previously reported (Hailer and Leonard 2008; Koblmüller et al. 2009; Wheeldon and White 2009; Kays et al. 2010).

The contemporary hybrid species-complex represents various forms. Specifically, a spectrum of coyote to eastern wolf to gray wolf phenotypes exists in a range of natural to human-modified landscapes, including regional differences in wolves (Mech and Paul 2008) and eastern coyotes (Kays et al. 2010). These differences demonstrate the range of hybrid forms likely resulting from a combination of differential population histories, disproportionate contributions from parental *Canis* species (Rutledge et al. 2010c), and potentially adaptive divergence on ecological factors, such as prey type. As a result, standard taxonomic nomenclature is difficult to apply to the classification, conservation, and management of wolves and coyotes in eastern North America. We encourage managers and policy makers to consider the evolutionary potential of these hybrid genomes because they may support the adaptability necessary to refill the ecological role once occupied by the purer wolf species that existed prior to European colonization. However, we also recognize that in situations where sufficient habitat exists for recolonization of historic species, efforts to minimize anthropogenic factors that exacerbate hybridization are an important aspect of conservation.

Assuming a three-species model of *C. lycaon*, *C. latrans*, and *C. lupus*, comparing the distributional patterns of Y-chromosomes and mtDNA revealed evidence of extensive multispecies hybridization across the eastern distribution, and the patterns were contrasted in different geographic regions at the population-level. In areas with previously described hybridizing wolves, such as northern Ontario, Manitoba, and Quebec (Grewal et al. 2004; Wheeldon and White 2009), Y-chromosomes from both wolf species were found and coyote Y-chromosomes were notably absent. Wolves in Quebec and northeastern Ontario had some coyote mtDNA, consistent with gene flow from Algonquin Park wolves (Grewal et al. 2004; Wilson et al. 2009). Despite an absence of gray wolf mtDNA in eastern coyotes, there was a surprisingly high frequency of gray wolf-like Y-chromosomes in eastern coyotes that were different from the haplotypes found in northern gray wolves in our study. This may reflect an origin of introgression related to the declining Plains wolves (*C. lupus nubilus*) or alternatively, these Y-chromosomes may have originated from dogs, as the majority of the Z*fy*-2 haplotypes in eastern coyotes are common in dog breeds (Sundqvist et al. 2006). The presence in eastern coyotes of Y-chromosome haplotypes observed in gray wolves but not dogs (i.e., 2CE, 2CF) certainly supports some level of gray wolf introgression, possibly via an eastern wolf conduit as coyotes expanded east through Ontario (Kays et al. 2010).

Although we cannot exclude the possibility that the eastern wolf originated from a more complex Pleistocene or early Holocene interaction of gray wolves and coyotes in eastern North America, overall, the sequence divergence and eastern-specificity of *Zfy-4* haplotypes suggests a longer standing history of an eastern North American evolved wolf, and the majority of genetic markers evaluated to date suggest a closer relationship of *C. lycaon* to a North American coyote lineage than the gray wolf lineage. As the Plains wolf has been extirpated and there is apparent Y-chromosome haplotype sharing between European gray wolves and dogs (i.e., FF and HT: Sundqvist et al. 2006; Sundqvist et al. 2001), these alternatives cannot be tested with our data set. Increasing representative data sets from nonhybridizing *Canis* populations, historic samples, and increased genomic surveys will facilitate the ability to reconstruct these population and species histories.

## Supporting Information

**Figure S1.** Eastern Wolf from Algonquin Provincial Park. Photo by Michael Runtz.

**Table S1.** Y-chromosome haplotypes observed in this study: haplotype codes correspond to the *Zfy* intron sequence followed by the allele letter designations for loci MS34 (first letter) and MS41 (second letter). Allele sizes of haplotypes were compared with those from previous studies (17 [Hxx], 24 [#], 25 [X]) to identify matching haplotypes and their corresponding locations: Nebraska (NE), Texas (TX), Alaska (AK), Northwest Territories (NWT), Saskatchewan (SK), Manitoba (MB), Northwestern Ontario (NWON), Northeastern Ontario (NEON), Algonquin Provincial Park (APP), Southeast Ontario (SEON), New York (NY), North Carolina (NC), Louisiana (LA), Maine/New Brunswick (ME/NB), Quebec (QC), and captive red wolves (RU).

## References

[b1] Adams JR, Leonard JA, Waits LP (2003). Widespread occurrence of a domestic dog mitochondrial DNA haplotype in southeastern US coyotes. Mol. Ecol.

[b2] Bandelt H-J, Forster P, Röhl A (1999). Median-joining networks for inferring intraspecific phylogenies. Mol. Biol. Evol.

[b3] Fain SR, Straughan DJ, Taylor BF (2010). Genetic outcomes of wolf recovery in the western Great Lakes states. Conserv. Genet.

[b4] Grewal SK, Wilson PJ, Kung TK, Shami K, Theberge MT, Theberge JB (2004). A genetic assessment of the eastern wolf (*Canis lycaon*) in Algonquin Provincial Park. J. Mammal.

[b5] Hailer F, Leonard JA (2008). Hybridization among three native North American *Canis* species in a region of natural sympatry. PLoS ONE.

[b6] vonHoldt BM, Pollinger JP, Earl DA, Knowles JC, Boyko AR, Parker H (2011). A genome-wide perspective on the evolutionary history of enigmatic wolf-like canids. Genome Res.

[b7] Kays R, Curtis A, Kirchman JJ (2010). Rapid adaptive evolution of northeastern coyotes via hybridization with wolves. Biol. Lett.

[b8] Koblmüller S, Nord M, Wayne RK, Leonard JA (2009). Origin and status of the Great Lakes wolf. Mol. Ecol.

[b9] Leonard JA, Wayne RK (2008). Native Great Lakes wolves were not restored. Biol. Lett.

[b10] Leonard JA, Vilà C, Wayne RK (2005). Legacy lost: genetic variability and population size of extirpated US grey wolves (*Canis lupus*. Mol. Ecol.

[b11] Librado P, Rozas J (2009). DnaSP v5: A software for comprehensive analysis of DNA polymorphism data. Bioinformatics.

[b12] Mech LD, Paul WJ (2008). Wolf body mass cline across Minnesota related to taxonomy?. Can. J. Zool.

[b13] Nowak RM (1979). North American Quaternary Canis. Monogr. Mus. Nat. Hist.

[b14] Nowak RM, Federoff NE (1998). Validity of the red wolf: response to Roy et al. Conserv. Biol.

[b15] Parker G (1995). Eastern coyote: the story of its success.

[b16] Pilgrim KL, Boyd DK, Forbes SH (1998). Testing for wolf-coyote hybridization in the Rocky Mountains using mitochondrial DNA. J. Wildl. Manage.

[b18] R Development Core Team (2011). R: A language and environment for statistical computing. http://www.R-project.org/.

[b19] Roy MS, Geffen E, Smith D, Ostrander EA, Wayne RK (1994). Patterns of differentiation and hybridization in North American wolflike canids, revealed by analysis of microsatellite loci. Mol. Biol. Evol.

[b20] Rutledge LY, Patterson BR, White BN (2010a). Analysis of *Canis* mitochondrial DNA demonstrates high concordance between the control region and ATPase genes. BMC Evol. Biol.

[b21] Rutledge LY, Bos KI, Pearce RJ, White BN (2010b). Genetic and morphometric analysis of sixteenth century *Canis* skull fragments: implications for historic eastern and gray wolf distribution in North America. Conserv. Genet.

[b22] Rutledge LY, Garroway CJ, Loveless KM, Patterson BR (2010c). Genetic differentiation of eastern wolves in Algonquin Park despite bridging gene flow between coyotes and grey wolves. Heredity.

[b23] Shaw C, Wilson P, White B (2003). A reliable molecular method of gender determination for mammals. J. Mammal.

[b17] Sundqvist AK, Ellegren H, Olivier M, Vilà C (2001). Y chromosome haplotyping in Scandinavian wolves (*Canis lupus*) based on microsatellite markers. Mol. Ecol.

[b24] Sundqvist AK, Bjornerfeldt S, Leonard JA, Hailer F, Hedhammar A, Ellegren H (2006). Unequal contribution of sexes in the origin of dog breeds. Genetics.

[b25] Wayne RK, Jenks SM (1991). Mitochondrial DNA analysis implying extensive hybridization of the endangered red wolf. Nature.

[b26] Wayne RK, Roy MS, Gittleman JL (1998). Response to Nowak and Federoff and Gardener. Conserv. Biol.

[b27] Wheeldon TJ, White BN (2009). Genetic analysis of historic western Great Lakes region wolf samples reveals early *Canis lupus**lycaon* hybridization. Biol. Lett.

[b28] Wheeldon TJ, Patterson BR, White BN (2010). Sympatric wolf and coyote populations of the western Great Lakes region are reproductively isolated. Mol. Ecol.

[b29] Wilson PJ, Grewal S, Lawford ID, Heal JNM, Granacki AG, Pennock D (2000). DNA profiles of the eastern Canadian wolf and the red wolf provide evidence for a common evolutionary history independent of the gray wolf. Can. J. Zool.

[b30] Wilson PJ, Grewal S, McFadden T, Chambers RC, White BN (2003). Mitochondrial DNA extracted from eastern North American wolves killed in the1800s is not of gray wolf origin. Can. J. Zool.

[b31] Wilson PJ, Grewal SK, Mallory FF, White BN (2009). Genetic characterization of hybrid wolves across Ontario. J. Hered.

