# Supplementary figures and images for: Y-chromosome evidence supports widespread signatures of three-species Canis hybridization in eastern North America
Source: Ecol Evol. 2012 Aug 13;2(9):2325–32. doi: 10.1002/ece3.301 (PMC3488682; doi:10.1002/ece3.301)

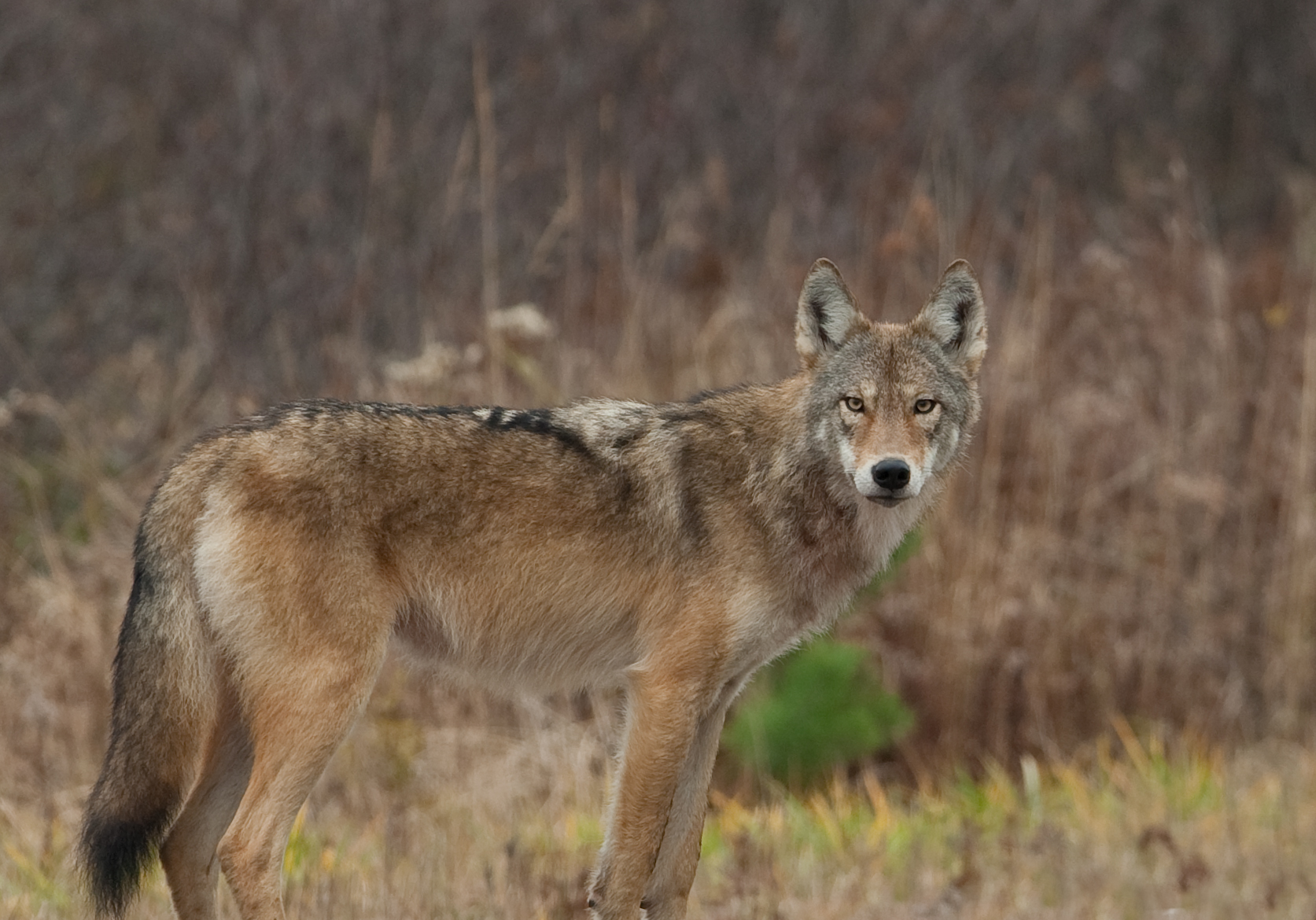

Supplement: Supplementary file 1 [file ece30002-2325-SD1.jpg]
