# Supplementary material for: Y-chromosome evidence supports widespread signatures of three-species Canis hybridization in eastern North America
Source: Ecol Evol. 2012 Aug 13;2(9):2325–32. doi: 10.1002/ece3.301 (PMC3488682; doi:10.1002/ece3.301)
Supplement: Supplementary file 2 [file ece30002-2325-SD2.doc]

**Table S1:** Y-chromosome haplotypes observed in this study: haplotype codes correspond to the *Zfy* intron sequence followed by the allele letter designations for loci MS34 (first letter) and MS41 (second letter). Allele sizes of haplotypes were compared to those from previous studies [17 (Hxx), 24 (#), 25 (X)] to identify matching haplotypes and their corresponding locations: Nebraska (NE), Texas (TX), Alaska (AK), Northwest Territories (NWT), Saskatchewan (SK), Manitoba (MB), Northwestern Ontario (NWON), Northeastern Ontario (NEON), Algonquin Provincial Park (APP), Southeast Ontario (SEON), New York (NY), North Carolina (NC), Louisiana (LA), Maine/New Brunswick (ME/NB), Quebec (QC), and captive red wolves (RU).

| Haplotype | Intron | Microsatellite | MS34a | MS34b | MS41a | MS41b | Haplotype | Location |
| --- | --- | --- | --- | --- | --- | --- | --- | --- |
| 1AQ | 1 | AQ | 172 | 180 | 212 | 218 | H7 | NE, SK |
| 1CD | 1 | CD | 172 | 178 | 214 | 210 |  | APP, SEON, NY, ME/NB |
| 1CI | 1 | CI | 172 | 178 | 214 | 214 | H20 | NE, SEON, NC, SK |
| 1CK | 1 | CK | 172 | 178 | 214 | 216 | H21 | NE, SK |
| 1CM | 1 | CM | 172 | 178 | 214 | 218 | H22 | NE, NC, SK, LA |
| 1CN | 1 | CN | 172 | 178 | 212 | 224 |  | SK |
| 1CO | 1 | CO | 172 | 178 | 212 | 220 | H3 | NE, SK |
| 1CP | 1 | CP | 172 | 178 | 212 | 222 | H4 | NE, TX |
| 1CQ | 1 | CQ | 172 | 178 | 212 | 218 |  | SK |
| 1CR | 1 | CR | 172 | 178 | 212 | 216 |  | APP |
| 1DQ | 1 | DQ | 172 | 176 | 212 | 218 |  | SK |
| 1GP | 1 | GP | 176 | 180 | 212 | 222 |  | APP, SEON, NY, ME/NB |
| 2AF | 2 | AF | 172 | 180 | 208 | 222 | H38 | NWT, MB, NWON, NEON |
| 2AT | 2 | AT | 172 | 180 | 208 | 220 |  | MB |
| 2CC | 2 | CC | 172 | 178 | 208 | 214 | H32 | AK, NWT, NWON, QC |
| 2CE | 2 | CE | 172 | 178 | 208 | 216 | H33 | AK, NWT, MB, NWON, NEON, QC, APP, SEON |
| 2CF | 2 | CF | 172 | 178 | 208 | 222 | H50 | NWT, NEON, QC, NY |
| 2CG | 2 | CG | 172 | 178 | 208 | 224 | H35 | NWT, APP |
| 2CS | 2 | CS | 172 | 178 | 208 | 226 | H36 | AK, NWT, NWON, QC, APP |
| 2CT | 2 | CT | 172 | 178 | 208 | 220 | H34 | NWT, NEON |
| 2DC | 2 | DC | 172 | 176 | 208 | 214 | H30 | AK, NWT, MB |
| 2EF | 2 | EF | 176 | 178 | 208 | 222 | H52, 7, G | NWT, APP |
| 2FF | 2 | FF | 174 | 178 | 208 | 222 | 12, L | NY, ME/NB |
| 2FG | 2 | FG | 174 | 178 | 208 | 224 | 71 | ME/NB |
| 2FL | 2 | FL | 174 | 178 | 208 | 218 | H1, 39 | RU, SEON, ME/NB, LA |
| 2HG | 2 | HG | 174 | 176 | 208 | 224 | 17 | NC |
| 2HS | 2 | HS | 174 | 176 | 208 | 226 | 18 | SEON, NC, LA |
| 2HT | 2 | HT | 174 | 176 | 208 | 220 | 33, Q | SEON, NY, LA |
| 3EA | 3 | EA | 176 | 178 | 212 | 212 | H15 | RU, TX, SK |
| 3EC | 3 | EC | 176 | 178 | 208 | 214 | H2 | TX |
| 3EJ | 3 | EJ | 176 | 178 | 212 | 214 |  | SK |
| 3EO | 3 | EO | 176 | 178 | 212 | 220 | H16 | TX, SK |
| 3FA | 3 | FA | 174 | 178 | 212 | 212 |  | SK |
| 3HI | 3 | HI | 174 | 176 | 214 | 214 |  | TX |
| 3HN | 3 | HN | 174 | 176 | 212 | 224 | H11 | TX |
| 3HO | 3 | HO | 174 | 176 | 212 | 220 | H10 | TX |
| 3HP | 3 | HP | 174 | 176 | 212 | 222 |  | TX |
| 3IQ | 3 | IQ | 174 | 180 | 212 | 218 |  | TX |
| 4AA | 4 | AA | 172 | 180 | 212 | 212 |  | NWON, QC, APP, SEON, NY, ME/NB |
| 4BB | 4 | BB | 170 | 182 | 212 | 226 |  | MB, NWON, NEON, QC, APP, SEON, LA |
| 4BR | 4 | BR | 170 | 182 | 212 | 216 |  | NC, LA |
